# Icariin Intervenes in Cardiac Inflammaging through Upregulation of SIRT6 Enzyme Activity and Inhibition of the NF-Kappa B Pathway

**DOI:** 10.1155/2015/895976

**Published:** 2015-01-20

**Authors:** Yang Chen, Tao Sun, Junzhen Wu, Bill Kalionis, Changcheng Zhang, Ding Yuan, Jianhua Huang, Waijiao Cai, Hong Fang, Shijin Xia

**Affiliations:** ^1^Center of Disease Prevention and Treatment, Shanghai Guanghua Hospital of Integrative Traditional Chinese and Western Medicine, Shanghai 200052, China; ^2^Department of Geriatrics, Shanghai Institute of Geriatrics, Huadong Hospital, Fudan University, Shanghai 200040, China; ^3^Department of Perinatal Medicine, Pregnancy Research Centre and University of Melbourne Department of Obstetrics and Gynaecology, Royal Women's Hospital, Parkville, VIC 3052, Australia; ^4^Medical College, China Three Gorges University, Yichang 443002, China; ^5^Key Laboratory of Cellular and Molecular Biology, Huashan Hospital, Fudan University, Shanghai 200040, China; ^6^Department of Diseases Prevention and Healthcare, Longhua Hospital, Shanghai University of Traditional Chinese Medicine, Shanghai 200032, China

## Abstract

The aim of the study was to investigate the effect of icariin (ICA) on cardiac aging through its effects on the SIRT6 enzyme and on the NF-*κ*B pathway. Investigating the effect of ICA on the enzymatic activity of histone deacetylase SIRT6 revealed a concentration of 10^−8^ mol/L ICA had a maximum activating effect on histone deacetylase SIRT6 enzymatic activity. Western analysis showed that ICA upregulated SIRT6 protein expression and downregulated NF-*κ*B (p65) protein expression in animal tissues and cell models. ICA upregulated the expression of SIRT6 and had an inhibitory effect on NF-*κ*B inflammatory signaling pathways as shown by decreasing mRNA levels of the NF-*κ*B downstream target genes TNF-*α*, ICAM-1, IL-2, and IL-6. Those effects were mediated directly or indirectly by SIRT6. We provided evidence that inflammaging may involve a novel link between the effects of ICA on SIRT6 (a regulator of aging) and NF-*κ*B (a regulator of inflammation).

## 1. Introduction

Icariin is an important, active component in* Herba Epimedii*. This well-known Chinese herbal medicine has proven efficacy in treating cardiovascular diseases and osteoporosis and in improving sexual and neurological function and is widely used to treat particular age-related diseases in oriental countries [1, 2]. Total flavone of* Epimedium* (TFE) is generally considered to be the active compound found in* Herba Epimedii*. Previous studies found that TFE prolongs the lifespan and has strong effects in 2BS cells [3],* C. elegans* [4], and* Drosophila* [5]. In addition, urine and plasma metabonomic studies showed TFE treatment altered age-related changes in aging rats to levels consistent with younger rats [6, 7]. Icariin represents an important active component in TFE and exhibits similar life prolonging effects of TFE in* C. elegans* [8] and mice. A significant feature of the natural aging process is that body function decline is chronic and progressive; therefore the desired intervention drug must have a therapeutic effect as well as being safe, have nontoxic side effects, and be suitable for long-term use. ICA fulfills these requirements as an aging intervention drug and has been studied extensively over many years.

Worldwide, research on delaying aging has a major focus on the silent information regulation protease family of Sirtuins [9]. Sirtuins belong to the Sir2 family of NAD(+)-dependent deacetylases and they regulate longevity in yeast,* C. elegans*, and* Drosophila*. In mammals, there are seven homologs of the yeast Sir2, Sirt1-7 [10, 11]. Current research on antiaging focuses mainly on SIRT1 and SIRT6 [12, 13]. There is strong experimental evidence to support that SIRT6, not SIRT1, is the enzyme responsible for prolonging the lifespan of higher organisms. SIRT6-deficient mice are small and at 2-3 weeks of age develop abnormalities that include profound lymphopenia, loss of subcutaneous fat, lordokyphosis, and severe metabolic defects. These mice eventually die after about 4 weeks. An important function of SIRT6 is to promote normal DNA repair, and SIRT6 loss leads to abnormalities in mice that have similarities with aging-associated degenerative processes [14]. Moreover, other research suggests that SIRT6 maintains genomic stability, and SIRT6 regulates glucose and fat metabolism, thereby inhibiting the inflammatory response. In the near future, SIRT6 research is expected to lead to new therapies for neurodegenerative diseases, metabolic diseases, and premature aging [15, 16].

Researchers are increasingly linking aging and inflammation, and they are focusing their attention on the NF-kappa B (NF-*κ*B) signaling pathway [17], which is a conserved signaling pathway that is active in the host immune response system. Not only does the NF-*κ*B signaling pathway regulate inflammation, oxidative stress and genotoxic stress, but it is also involved in the self-regulation of homeostatic functions such as apoptosis, autophagy, and tissue atrophy. SIRT1, SIRT6, and FOXO genes are important genes that can inhibit inflammaging caused by the NF-*κ*B signaling pathway [18]. NF-*κ*B is a stress-responsive transcription factor that induces expression of target genes involved in aging-related processes including cell senescence, apoptosis, and inflammation. NF-*κ*B signaling is regulated by SIRT6, which is recruited to NF-*κ*B target gene promoters by physical interaction with the NF-*κ*B (P65) subunit RELA. SIRT6 deacetylates histone H3 lysine 9 on target gene promoters, thereby altering chromatin structure to facilitate NF-*κ*B destabilization and signal termination [19].

Numerous studies suggest that ICA can inhibit the NF-*κ*B signaling pathway and reduce the body's inflammatory response. Given the evidence that ICA can delay aging and prolong average and maximum life expectancy, it is uncertain whether ICA acts on SIRT6 and the NF-*κ*B signaling pathways. Thus, the aim of the study was to provide experimental evidence that ICA acts through SIRT6 and the NF-*κ*B signaling pathway to prevent inflammaging.

## 2. Materials and Methods

### 2.1. Animals

Healthy male BALB/c mice that were specific pathogen-free (SPF) and 8~9 weeks of age were provided by Vital River Laboratory Animal Technology Co. Ltd. All procedures for handling animals were approved by the Animal Experimentation Committee of Laboratory Animal Center of Shanghai Public Health Clinical Center. 23 mice were fed to 12 months of age in single cage and then randomly divided into a normal control group (*n* = 11, fed by feed pellets) and an ICA-treated group (*n* = 12, fed by feed pellets contained in 0.02% ICA for 3 months when the mice were 21 months old). Three-month old mice were used as the young control group (*n* = 10), and when mice were 24 months of age they were used as the aged group (*n* = 10). Mice were fed* ad libitum*, and tissue specimens from the heart were taken for testing.

### 2.2. Chemicals and Reagents

ICA (98.5% pure and provided by Shanghai Second Military Medical University) was used for the preparation of mouse feed. Mouse feed was mixed with ICA powder, dried at 70~90°C, sterilized, and then prepared into feed pellets. The NF-*κ*B inhibitor pyrrolidine dithiocarbamate (PDTC), 1-methyl-3-isobutyl-xanthine (IBMX), dexamethasone, synthetic human insulin, and SIRT6 enzyme inhibitor nicotinamide were purchased from Sigma (USA). High glucose DMEM medium and fetal bovine serum were purchased from Gibco. Trizol was purchased from Invitrogen. Real-time PCR kits were purchased from TaKaRa (Japan). Rabbit anti-SIRT6 was purchased from Abcam (UK). Mouse-specific NF-*κ*B (p65) antibody was from Cell Signal Technology (USA) and HRP-conjugated anti-rabbit secondary antibody was purchased from Beyotime (Shanghai, China).

### 2.3. Protocol for Murine Aortic Endothelial Cell (ECs) Culture

Three-month old murine aortic ECs were isolated and cultured by enzymatic dissociation as previously described [20, 21]. Briefly, the full length of the thoracic aorta was removed from mice under sterile conditions, rinsed 3 times with phosphate-buffered saline solution (PBS), and placed into a 100-mm culture dish (Corning, NY, USA) filled with serum-free Dulbecco modified Eagle medium (DMEM, Hyclone, Logan, UT) on ice. The vessel was gently cleaned of periadventitial fat and connective tissue. The thoracic aorta was opened and rinsed with serum-free DMEM, placed intimal side down on a sterile plate containing 0.2% collagenase type I (Sigma, St. Louis, MO), and incubated at 37°C for 30 min. Cells were gathered by gentle abrasion with a cell scraper (Costar, Pleasanton, CA) from the edges to the center of the specimen and then rinsed with 5 mL of DMEM plus serum to arrest the digestion process. The medium containing detached ECs was collected and the cell suspension was centrifuged at 2000 rpm for 10 minutes. The cell pellet was washed twice, suspended in DMEM supplemented with 10% fetal bovine serum (FBS, Hyclone), 100 U/mL penicillin, 100 *μ*g/mL streptomycin, and 75 *μ*g/mL endothelial cell growth supplement (sigma, USA), and placed into a 60-mm culture dish (Corning, USA). The dish was then placed in a humidified incubator at 37°C with 5% CO_2_. Thereafter, the medium was changed every other day. Once the cells had formed a monolayer, the cells were subcultured. More than 95% of the cells were positive for von Willebrand factor antibody (sc-59810, Santa Cruz, USA) staining (data not shown).

### 2.4. Detection of Senescence-Associated *β*-Galactosidase (SA-*β*-Gal)* In Vitro*

Cell Grouping: Three-month old murine aortic ECs were divided into three groups: normal, TNF-*α*-treated, and TNF-*α*-treated with ICA intervention. In the TNF-*α*-treated group, exogenous TNF-*α* (10 ng/mL) was added into cell culture solution for 36 hours. Subsequently, TNF-*α* (10 ng/mL) was added again for 36 hours to the TNF-*α*-treated group. In the TNF-*α*-treated with ICA intervention group, exogenous TNF-*α* (10 ng/mL) and ICA (10^−8 ^mol/L) were simultaneously added into cell culture solution for 36 hours, and then TNF-*α* (10 ng/mL) was added separately for 36 hours.

A senescence-associated *β*-galactosidase (SA-*β*-gal) staining kit (Beyotime, Shanghai) was used according to the manufacturer's protocol. Briefly, cells were seeded at subconfluence in a 6-well plate and further incubated in serum-free medium for 24 hours. Cells were then treated according to the above groups. After 72 h, cells were washed twice with PBS; 1 mL X-gal dye was added into each well and then incubated at 37°C, 5% CO_2_ for 4 hours. The percentage of SA-*β*-gal-positive cells was determined by counting the number of blue cells under bright-field illumination and the total number of cells in the same field under phase contrast. At least 5 random fields were counted for each culture dish.

### 2.5. Administration Aortic Endothelial Cells (ECs) Experiments

Young murine aortic ECs were divided into seven groups: (1) young treated with PBS, (2) young treated with DMSO, (3) young + TNF-*α*, (4) young + TNF-*α* + ICA (10^−8^ mol/L), (5) young + TNF-*α* + ICA + PDTC, (6) young + TNF-*α* + ICA + Nicotinamide, and (7) young + TNF-*α* + ICA + PDTC + Nicotinamide. Groups 1 and 2 were controls treated with PBS and DMSO, respectively, for 12 hours. Groups 3–6 were all treated with exogenous TNF-*α* (10 ng/mL for 30 min to generate mild inflammation). Treatments with ICA, PDTC (final concentration of PDTC is 10 *μ*M), and Nicotinamide (final concentration of Nicotinamide is 50 mM) were for 12 hours.

### 2.6. SIRT6 Enzyme Activity Test* In Vitro*

ICA (molecular weight 676.65) was dissolved in DMSO to prepare different working concentrations as follows: 10^−4^, 10^−6^, 10^−8^, 10^−10^, 10^−12^, 10^−14^, and 10^−16^ mol/L. The SIRT6 Direct Fluorescent Screening Assay Kit (Cayman Chemical Company, USA) was used to test the activation effect of ICA on SIRT6 enzymatic activity. The assay was carried out according to the manufacturers' instructions. The assay wells comprised 100% initial enzyme activity wells (added SIRT6 enzyme and DMSO), background wells (added DMSO alone), and sample wells (added SIRT6 enzyme and different ICA concentrations). The plates were read within 30 min using a microplate reader (TECAN, USA) with an excitation wavelength of 350–360 nm and an emission wavelength of 450–460 nm. The background well values were subtracted from the 100% initial activity wells and from the sample wells. The following equation was used to calculate the percent activation. %Activation = [(initial activity − sample)/initial activity] × 100.

### 2.7. Western Blot Analysis

Murine heart tissue protein was extracted according to the manufacturer's instructions. Protein concentrations were measured using a BCA Protein Assay (Rockford, IL). Equal amounts of protein were loaded onto 10%–15% SDS-PAGE gels, transferred to nitrocellulose (NC) membranes, and blocked with 5% nonfat milk in TBST buffer (0.121% Tris-HCl, 0.9% NaCl, and 0.1% Tween-20, PH7.5) for 1 hour. Membranes were incubated with a 1 : 1000 dilution of primary SIRT6 antibody or a 1 : 2000 dilution of NF-*κ*B (p65) antibody. After washing, the blots were incubated with a 1 : 2000 dilution of horseradish peroxidase- (HRP-) labeled IgG secondary antibody for 1 hour. Signals on the blots were then developed using the enhanced chemiluminescence (ECL) system and analyzed by an Odyssey Imager (Li-COR Biosciences, USA). Densitometric analysis was performed with ImageJ software (National Institute of Health, Bethesda, MD, USA).

### 2.8. Reverse Transcription and Semiquantitative Real-Time Polymerase Chain Reaction Assay

Lysed murine aortic ECs were transferred into a tube containing Trizol, and total RNA was isolated according to the manufacturer's protocol. Total RNA was used for cDNA synthesis and the RT-PCR was carried out according to the manufacturer's instructions. The PCR was performed in a commercial real-time thermocycler (ABI Prism 7000, Applied Biosystems, Germany) using the following cycle parameters: a 94°C predenaturation for 3 min, followed by 35 cycles of 94°C for 40 seconds, 56°C for 50 seconds, and 72°C for 90 seconds, and an extension cycle of 70°C for 10 min. The target gene transcripts in each sample were normalized to the levels of human GAPDH (glyceraldehydes-3-phosphate dehydrogenase). The primers used were as follows:

 SIRT6
 forward, 5′-AGGCCGTCTGGTCATTGTC-3′,
 reverse, 5′-GCACATCACCTCATCCACGTA-3′;
 TNF-*α*
 forward, 5′-GCACCACCATCAAGGACTCAAATG-3′,
 reverse, 5′-GACAGAGGCAACCTGACCACTC-3′;
 ICAM-1
 forward, 5′-GGAGACGCAGAGGACCTTAACAG-3′,
 reverse, 5′-CGACGCCGCTCAGAAGAACC-3′;
 IL-2
 forward, 5′-TCACCCTTGCTAATCACTCCTCAC-3′,
 reverse, 5′-TGCTGCTGCTGCTGCTGTG-3′;
 IL-6
 forward, 5′-AACCGCTATGAAGTTCCTCTC-3′,
 reverse, 5′-TCCTCTGTGAAGTCTCCTCT-3′;
 GAPDH
 forward, 5′-GGCACAGTCAAGGCTGAGAATG-3′,
 reverse, 5′-ATGGTGGTGAAGACGCCAGTA-3′.

### 2.9. Immunocytochemistry of NF-*κ*B (p65) Nuclear Translocation by Confocal Laser Scanning Microscopy

ECs were plated on glass coverslips and fixed in methanol and acetone (1 : 1) overnight at 4°C. After washing, samples were permeabilized for 15 min with buffer containing saponin (50 mm Tris, 200 mm NaCl, and 0.05% saponin, pH7.5) at room temperature. Cells were subsequently incubated with buffer (50 mm Tris, 200 mm NaCl, pH7.5) containing 3% goat serum for 30–60 min to block nonspecific binding. To identify cell nuclei, cells were stained with DAPI (5 *μ*g/mL) at room temperature for 5 minutes, and then washed with PBS three times. For NF-*κ*B (p65) staining, cells were incubated with the primary antibodies (NF-*κ*B antibody, 1 : 500 dilution) for 2 hours at 37°C. Next, the cells were washed three times and incubated with secondary antibody (anti-rabbit Cy3, 1 : 500 dilution) for 1 hours at 37°C. Cells were covered with Prolong mounting medium and Prolong antifade reagent (Invitrogen, USA) and stored at 4°C. Cells were mounted and examined under a confocal laser scanning microscope (Leica, Germany).

### 2.10. Relative Quantification Detection of NF-*κ*B (p65) Nuclear Translocation

NF-*κ*B (p65) relative nuclear translocation quantitative detection kit (FIVEphoton Biochemicals, USA) has no radioactivity and high sensitivity and can detect specific-binding between DNA sites and transcription factors in nuclear extract protein. According to the manufacturer's protocol, the specific double-stranded DNA (dsDNA) sequence containing the transcription factor NF-*κ*B (p65) response element was preplaced inside the 96-well plates. NF-*κ*B (p65) specific primary antibody (1 : 100 dilution) was added, followed by horseradish peroxidase-labeled secondary antibody (1 : 100 dilution). The absorbance was read at 450 nm on a microplate reader (TECAN, USA).

### 2.11. Statistical Analysis

Experiments were performed in triplicate. Each data point was presented as the mean ± standard deviation (SD). Comparisons between groups were analyzed using the *t*-test. Statistical significance was evaluated using one-way ANOVA with SPSS 13.0 software. A value of *P* < 0.05 was considered statistically significant.

## 3. Results

### 3.1. ICA Has an Activation Effect on SIRT6 Enzyme Activity* In Vitro*

**Figure 1:**
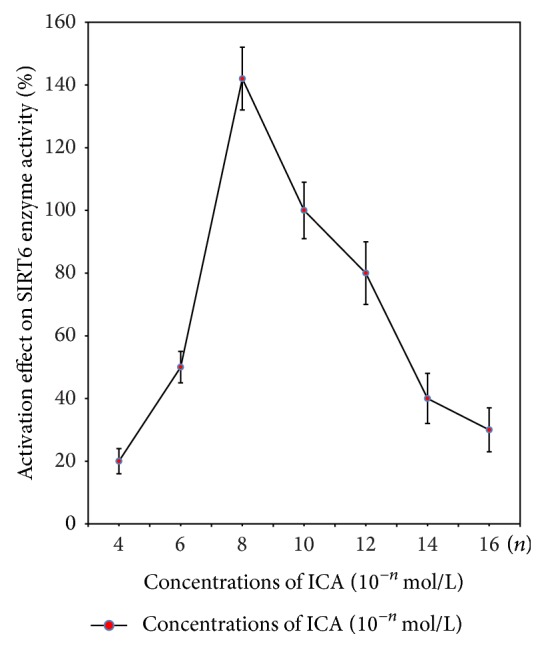
The effect of varying concentrations of ICA on the activation of SIRT6 enzyme activity. Concentrations of ICA were in 10^−*n*^ mol/L, where *n* = 4, 6, 8, 10, 12, 14, 16. The SIRT6 direct fluorescent screening assay was carried out as described in Section 2.6. ICA shows a low % activation effect on the enzymatic activity of SIRT6 in high concentrations of ICA (10^−4^ mol/L). With decreasing concentrations of ICA, the percent activation effect on SIRT6 enzyme activity increases and peaks at an ICA concentration of 10^−8^ mol/L.

As shown in Figure 1, ICA shows a low percent activation effect (20% ± 4%) on the SIRT6 enzyme activity at the highest ICA concentration (10^−4 ^mol/L). With decreasing concentrations of ICA, the activation effect on SIRT6 enzyme activity increased and reached a maximum (142% ± 10%) at a concentration of 10^−8 ^mol/L. An activation effect of ICA on SIRT6 enzyme activity was detected even at very low concentrations of ICA (10^−14^, 10^−16^ mol/L).

### 3.2. Experiments on Animals

#### 3.2.1. The Effect of ICA on SIRT6 Protein Expression in Heart Tissue

**Figure 2:**
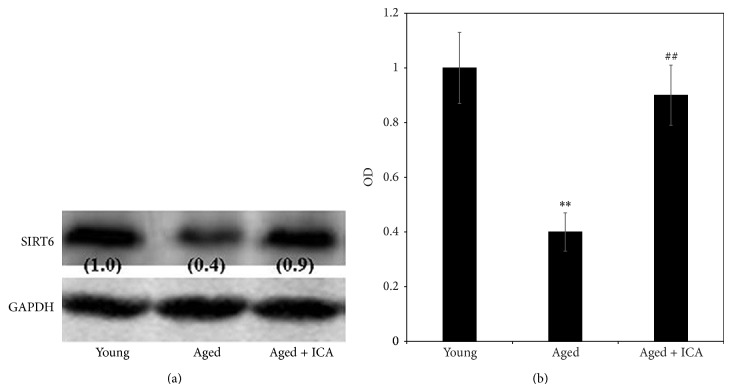
(a) shows a band of the expected size corresponding to SIRT6 protein in murine heart tissue extract prepared from 3-month old mice (young), 24-month old mice (aged), and 24-month old mice treated with ICA (aged + ICA). Numbers in brackets are the normalized OD (optical density) values. (b) shows the OD (optical density) comparison of SIRT6 protein expression in heart tissue (fold of control value). Note: aged group compared with the young group, ^**^ *P* < 0.01; aged group compared with the aged + ICA intervention group, ^##^ *P* < 0.01.

As shown in Figure 2, SIRT6 protein expression was detected in murine heart tissue isolated from the young, aged, and aged with ICA intervention groups. The results suggest that SIRT6 protein expression in aged mice hearts was decreased when compared with young mice. In the aged with ICA intervention group, SIRT6 protein expression was significantly upregulated in heart tissue compared with the aged group (Figure 2(b)).

#### 3.2.2. The Effect of ICA on NF-*κ*B (p65) Protein Expression in Heart Tissue

**Figure 3:**
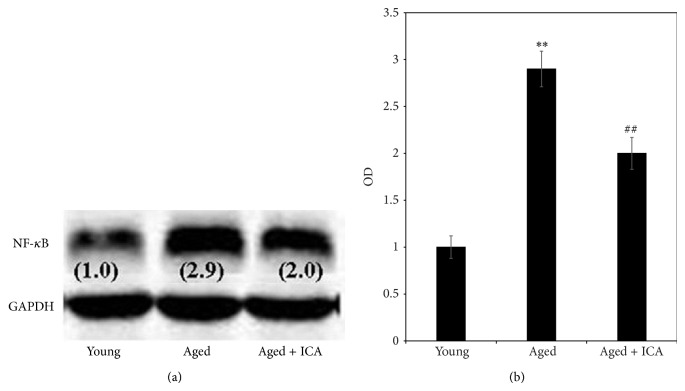
(a) shows a western analysis with a band of the expected size corresponding to NF-*κ*B (p65) protein in mouse heart tissue extract prepared from 3-month old mice (young), 24-month old mice (aged), and 24-month old mice treated with ICA (aged + ICA) (a). Numbers in brackets are the normalized OD (optical density) values. (b) shows the normalized OD value comparison of NF-*κ*B (p65) protein expression in heart tissue (fold increase compared with the control young group set at 1.0). Note: aged group compared with the young group, ^**^ *P* < 0.01; aged group compared with the aged group + ICA intervention group, ^##^ *P* < 0.05.

As shown in Figure 3, NF-*κ*B (p65) protein expression was detected mouse heart tissue isolated from the young, aged, and aged with ICA intervention groups. The results suggest NF-*κ*B (p65) protein expression was increased in aged mice hearts compared with young mice. In the aged with ICA intervention group, NF-*κ*B (p65) protein expression was decreased in heart tissue compared with the aged mice.

### 3.3. The Result of Detection of SA-*β*-Gal in* In Vitro* Mouse Aortic ECs

**Figure 4:**
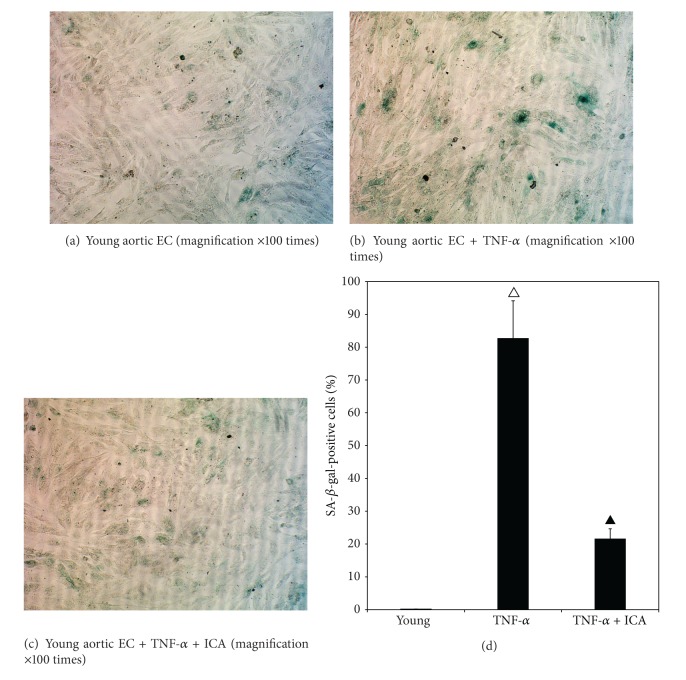
Detection of SA-*β*-Gal in mouse aortic ECs isolated from young mice (3 months old) and treated with TNF-*α* and ICA. (a) shows a microphotograph with SA-*β*-Gal stained cells under bright-field illumination. Very few blue cells were detected in the field, with the young control group. (b) shows that increased numbers of blue-stained SA-*β*-Gal cells were present in young + TNF-*α*-treated group. (c) shows a qualitative decrease in the number of blue-stained SA-*β*-Gal cells in the young + TNF-*α* + ICA intervention group compared with the young + TNF-*α* group. (d) shows the quantitation of the percentage of SA-*β*-gal-positive cells in all fields counted. Young + TNF-*α* compared with the young group, ^△^ *P* < 0.01; young + TNF-*α* + ICA compared with the young + TNF-*α*-treated group, ^▲^ *P* < 0.01.

Figure 4 shows that the percentage of SA-*β*-gal-positive cells in the TNF-*α*-treated young group was significantly higher (*P* < 0.01) compared with the young group. After the cells in normal group were treated with TNF-*α* and ICA, the percentage of blue-stained cells was significantly decreased (*P* < 0.01). These data show that TNF-*α* can induce endothelial cell senescence in young cells and the effect can be suppressed by ICA treatment.

### 3.4. The Results of Aortic Endothelial Cells (ECs) Experiments

#### 3.4.1. The Effect of ICA on SIRT6 and NF-*κ*B (p65) Protein Expression in Each Group of Aortic ECs

**Figure 5:**
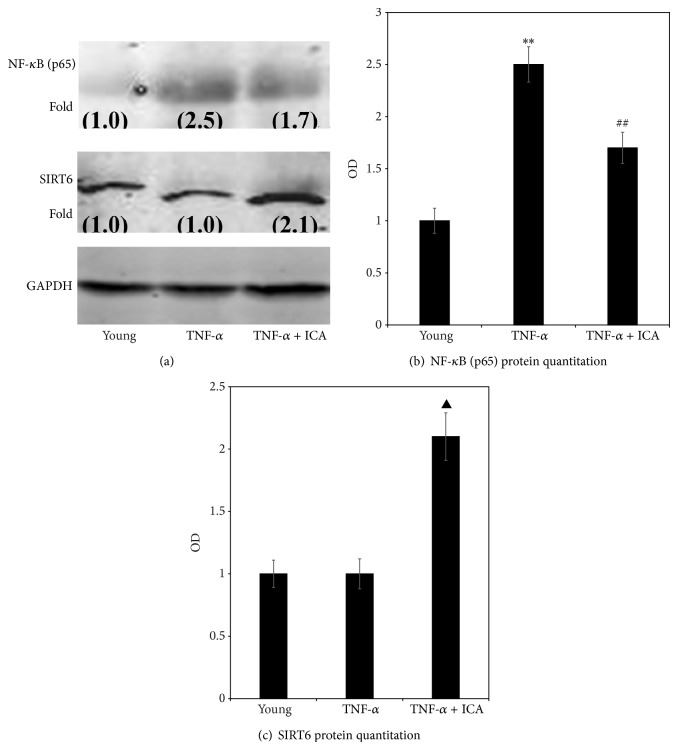
(a) shows a western analysis with band sizes corresponding to NF-*κ*B (p65) and SIRT6 protein, which were obtained from aortic ECs sampled in the young group (young), young treated with TNF-*α* group (+TNF-*α*), and young treated with TNF-*α* + ICA intervention group (TNF-*α* + ICA). Numbers in brackets are normalized OD (optical density) values, where the value of 1.0 was set for the young group. (b) shows OD comparison of NF-*κ*B (p65) protein expression in aortic ECs (fold increase over the young value set at 1.0). Compared with young cells, ^**^ *P* < 0.01; compared with young cells + TNF-*α*, ^##^ *P* < 0.01. (c) shows OD comparison of SIRT6 protein expression in aortic ECs (fold increase over the young value set at 1.0). Young + TNF-*α* + ICA compared with young + TNF-*α*, ^▲^ *P* < 0.01.

Figure 5(a) shows that after induction of mild inflammation in aortic endothelial cells by addition of exogenous TNF-*α*, NF-*κ*B (p65) protein expression was upregulated significantly, while Figure 5(b) shows there was no effect on SIRT6 protein expression. Addition of ICA to TNF-*α* treated cells significantly upregulated protein expression of SIRT6 (Figure 5(b)) while downregulating NF-*κ*B (p65) (Figure 5(a)).

#### 3.4.2. The Effect of ICA on mRNA Expression of SIRT6 and NF-*κ*B (p65) Downstream Inflammatory Cytokines

**Figure 6:**
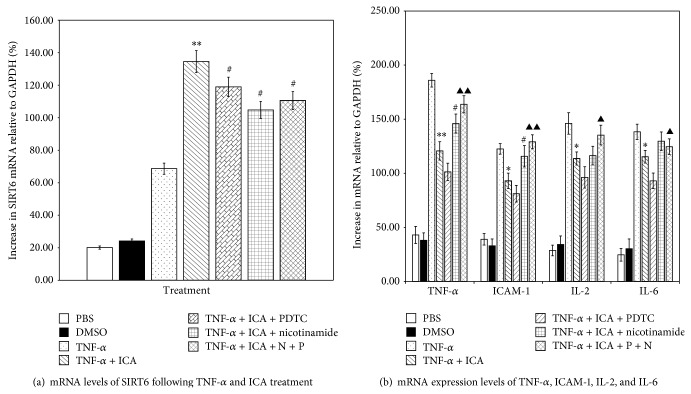
(a) Comparison of mRNA level of SIRT6 in different treatment groups. Young aortic ECs + PBS or + DMSO were controls. Various treatments of young aortic ECs are shown. After treatment with TNF-*α*, the mRNA expression level of SIRT6 was increased. Treatment with TNF-*α* and ICA resulted in a significant increase in SIRT6 mRNA relative to TNF-*α* treatment alone. TNF-*α* + ICA treated cells subsequently treated with PDTC or Nicotinamide or PDTC and Nicotinamide also showed increased SIRT6 mRNA levels; however there were no significant differences between these treatment groups and treatment with TNF-*α* + ICA. Note: TNF-*α* + ICA compared with TNF-*α* treated group, ^**^ *P* < 0.01; TNF-*α* + ICA + PDTC, TNF-*α* + ICA + Nicotinamide and TNF-*α* + ICA + PDTC(P) + Nicotinamide(N) compared with TNF-*α* + ICA group, ^#^ *P* > 0.05. (b) Comparison of mRNA levels of TNF-*α*, ICAM-1, IL-2, and IL-6 in different treatment groups. Young aortic ECs treated with PBS or DMSO were the negative controls. Various treatments of young aortic ECs are shown. When given an exogenous TNF-*α* stimulus, mRNA levels of TNF-*α*, ICAM-1, IL-2, and IL-6 were significantly increased in all treatment groups. Treatments TNF-*α* and ICA resulted in a significant reduction in mRNA levels compared with TNF-*α* treatment alone. Treatment with ICA + PDTC further reduced the mRNA levels of the four inflammatory genes. Treatment with ICA + Nicotinamide partially reversed the effect of ICA, with significant increases in TNF-*α* and ICAM-1 mRNA levels. Treatment with ICA + PDTC + Nicotinamide significantly increased mRNA levels of the four inflammatory genes compared with the TNF-*α* + ICA + PDTC treated group and completely reversed the effect of ICA on ICAM-1 levels. Note: TNF-*α* + ICA compared with TNF-*α* treated group, ^**^ *P* < 0.01 or ^*^ *P* < 0.05. TNF-*α* + ICA + Nicotinamide compared with TNF-*α* + ICA treated group, ^#^ *P* < 0.05. TNF-*α* + ICA + PDTC(P) + Nicotinamide(N) compared with TNF-*α* + ICA + PDTC treated group ^▲▲^ *P* < 0.01 or ^▲^ *P* < 0.05.

As shown in Figure 6(a), treatment with the proinflammatory cytokine TNF-*α* increased mRNA levels of SIRT6 compared with young control cells treated with PBS or DMSO. With the intervention of ICA, PDTC, Nicotinamide alone, or all together, compared to TNF-*α* group, there was a significant increase in SIRT6 mRNA level. The increase was highest in the ICA treatment group. There were no significant differences between the TNF-*α* + ICA + PDTC, TNF-*α* + ICA + Nicotinamide, or TNF-*α* + ICA + PDTC + Nicotinamide treated groups compared with the TNF-*α* + ICA treated group (*P* > 0.05).

In Figure 6(b) after addition of TNF-*α*, mRNA levels of NF-*κ*B (p65) downstream inflammatory cytokines (TNF-*α*, ICAM-1, IL-2, and IL-6) were increased compared with young control cells treated with PBS or DMSO. The increase in TNF-*α* mRNA levels was the highest. After the intervention with ICA, the increased mRNA levels of TNF-*α*, ICAM-1, IL-2, and IL-6 were all significantly decreased. The decrease in TNF-*α* mRNA levels was the greatest of the four cytokines tested (*P* < 0.01). When ICA and PDTC (NF-*κ*B nuclear translocation blocker) were added together, this resulted in a further decrease in mRNA levels of TNF-*α*, ICAM-1, IL-2, and IL-6. Nicotinamide treatment partially reversed the effect of ICA, with significant increases in TNF-*α* and ICAM-1 mRNA levels. Treatment with ICA + PDTC + Nicotinamide significantly increased mRNA levels of the four inflammatory genes compared with the TNF-*α* + ICA + PDTC treated group and completely reversed the effect of ICA on ICAM-1 levels.

#### 3.4.3. Qualitative Assessment of the Effect of ICA on NF-*κ*B (p65) Nuclear Translocation

**Figure 7:**
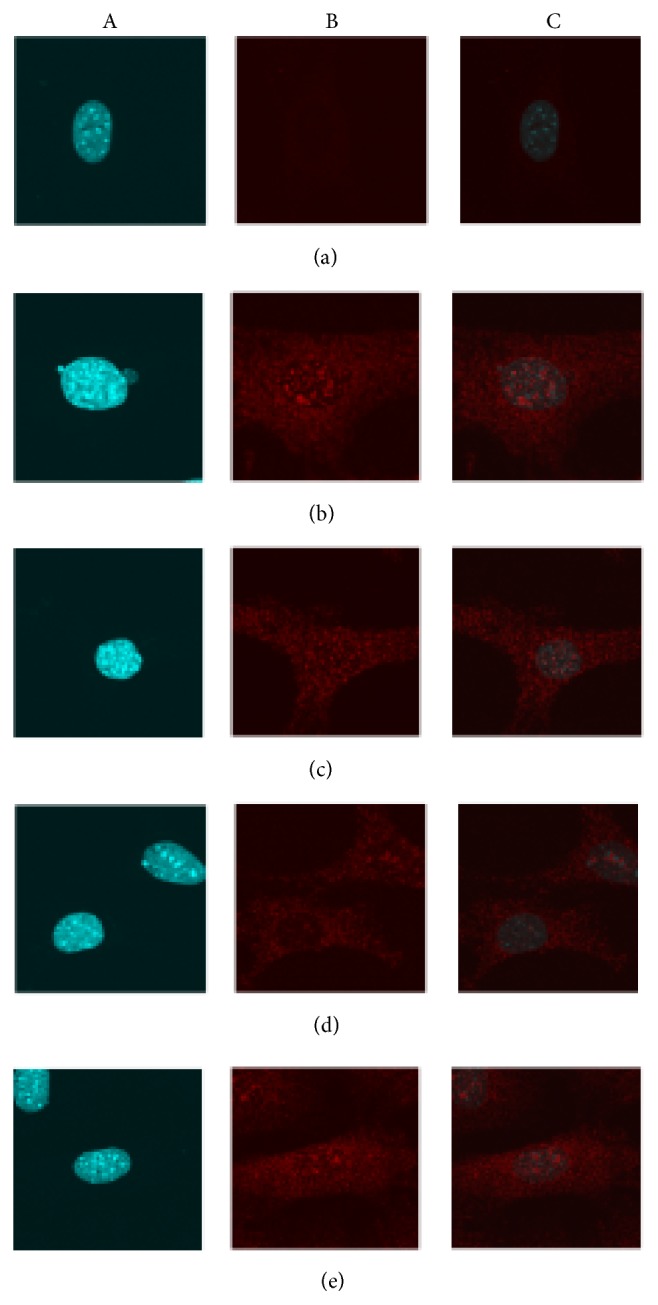
The effect of ICA on NF-*κ*B (p65) nuclear translocation was qualitatively assessed on young mouse aortic ECs. Magnification ×650 times. (a) shows the nuclei in aortic ECs by DAPI stain. (b) Red fluorescence signals (represent NF-*κ*B p65 protein) were shown in aortic ECs both including the areas of nuclei and cytoplasm. (c) The combination of figure A and figure B helps to determine the red fluorescence signal in the nuclei and thereby indirectly showing NF-*κ*B (p65) nuclear translocation from cytoplasm to nuclear. (a) Young aortic EC group (PBS and DMSO treatment). (b) Young + TNF-*α* group. (c) Young + TNF-*α* + ICA. (d) Young + TNF-*α* + ICA + PDTC. (e) Young + TNF-*α* + ICA + PDTC + Nicotinamide. As shown in the Figure 7(a), in the quiescent condition treatment with PBS or DMSO, very low red fluorescence signals were detected in aortic ECs and in the area of nuclei almost no red fluorescence was detected. After being treated by TNF-*α* to induce cell inflammation, red fluorescence signals were strongly detected in the cell, especially condensed in the nuclear location Figure 7(b). In the presence of ICA or ICA + PDTC intervention, such condensed red fluorescence signals in the nuclei were both reduced and ICA + PDTC intervention showed a stronger inhibitory effect than ICA, in Figures 7(c) and 7(d), respectively. As shown in Figure 7(e), adding Nicotinamide (SIRT6 enzyme inhibitor) red fluorescence signal became stronger than that in Figure 7(d).

NF-*κ*B (p65) is normally present in the cytoplasm in an inactive state.  Figure 7(a) shows that very low NF-*κ*B (p65) cytoplasmic protein was detected in young aortic ECs treated with PBS and DMSO. Following TNF-*α* treatment for 30 mins, NF-*κ*B (p65) protein is detected in the cytoplasm and at qualitatively increased levels in nucleus (Figure 7(b)). After TNF-*α* treatment, and intervention of ICA for 12 hours, NF-*κ*B (p65) protein decreases in the nucleus (Figure 7(c)) and qualitatively is further reduced upon addition of PDTC [specific inhibitor of NF-*κ*B (p65)] (Figure 7(d)). The NF-*κ*B (p65) signal in the nucleus is qualitatively increased when the Nicotinamide (SIRT6 inhibitor) is added in the presence of PDTC (Figure 7(e)).

#### 3.4.4. The Effect of ICA on NF-*κ*B (p65) Nuclear Translocation (Relative Quantification)

**Figure 8:**
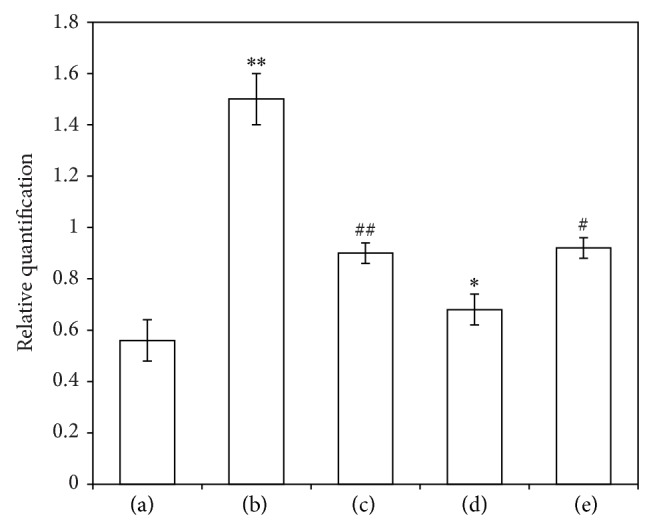
The effect of ICA on NF-*κ*B (p65) nuclear translocation (relative quantification). (a) Young aortic ECs group (PBS and DMSO treatment). (b) Young + TNF-*α* group. (c) Young + TNF-*α* + ICA. (d) Young + TNF-*α* + ICA + PDTC. (e) Young + TNF-*α* + ICA + PDTC + Nicotinamide. Note: compared with normal group, ^**^ *P* < 0.01; compared with TNF-*α* group, ^##^ *P* < 0.01; compared with young + TNF-*α* + ICA group, ^*^ *P* < 0.05; and compared with young + TNF-*α* + ICA + PDTC group, ^#^ *P* < 0.05.

Figure 8 shows that NF-*κ*B (p65) nuclear translocation relative in the young + DMSO group is 0.56 ± 0.08. After the addition of TNF-*α*, the relative value increased significantly to 1.5 ± 0.1 (*P* < 0.01). The relative values of the young + TNF-*α* + ICA (0.9 ± 0.04) were significantly decreased compared with the young + TNF-*α* group (*P* < 0.01). Upon addition of PDTC (young + TNF-*α* + ICA + PDTC group) levels were decreased (0.68 ± 0.06) relative to the young + TNF-*α* + ICA group (*P* < 0.05). Treatment with Nicotinamide reversed the effect of PDTC (*P* < 0.05).

## 4. Discussion

A concentration range of ICA from 10^−4^ to 10^−16^ mol/L was used in an* in vitro* assay to detect an activation effect on the enzyme activity of SIRT6. ICA showed a low activation effect on the enzymatic activity of SIRT6 at high concentrations of ICA (10^−4 ^mol/L) but, with decreasing concentrations of ICA, activation of SIRT6 enzyme activity occurred and reached the peak value at an ICA concentration of 10^−8^ mol/L. There was evidence for an activation effect of ICA on SIRT6 even at quite low drug concentrations of 10^−16^ mol/L. These data suggest that an ICA concentration of 10^−8 ^mol/L gives an optimal activation effect on the enzymatic activity of SIRT6. We cannot rule out the possibility that at high ICA concentrations (10^−4 ^mol/L) there is a substrate inhibition or a toxic effect on SIRT6 enzyme activity.

The natural aging process is associated with chronic, low-grade, mild inflammation. In 2000, Franceschi et al. found that the serum level of IL-6 is very low in young people but with aging to 60 years, the levels of IL-6 increased gradually. This phenomenon, called inflammaging (inflammation-aging) [22–24], and this condition are now thought to be critical in the onset of sarcopenia, frailty, and the pathogenesis of several age-related chronic diseases, such as Parkinson's and Alzheimer's disease (AD), atherosclerosis, and type 2 diabetes [25–29]. These diseases (and disabilities) appear to share the same process of inflammation, leading to the concept of an age-related “diseasome” [30]. Accordingly, genes and epigenetic factors involved in the regulatory pattern of inflammation are expected to play a role in human ageing.

Through receptor-mediation, inflammatory cytokines including IL, interferons, TNF, colony stimulating factor, and TGF can participate in the complex cell-cell regulatory network in the body through paracrine and/or autocrine effects. In some cases these factors enter the circulation, causing systemic effects. By interacting with each other, many inflammatory cytokines constitute the inflammatory cytokine network [31], which can be divided into two types: the proinflammatory cytokine network (TNF-*α*, IFN-2*γ*, IL-1, IL-6, and IL-8) and the anti-inflammatory cytokine network (TGF-*β*1, IL-4, IL-10, and IL-13).

Important physiological functions regulate the growth and differentiation of immune cells and the body's defense reaction. NF-*κ*B is an important factor that regulates and controls gene expression of many cytokines, cell adhesion molecules, chemotactic factors, and the acute response proteins. Following activation, NF-*κ*B can enhance the gene transcription levels of TNF-*α*, IL-1, IL-6, IL-8, ICAM-1, and other cytokines and thereby participate in the body's immune defense response [32–34].

The immune response is crucial for the body's normal physiological function. Inflammation can be of moderate benefit but is otherwise harmful. The change in the inflammatory cytokine network controls the direction of the development of inflammation. The dynamic counterbalance between the proinflammatory cytokine network and the anti-inflammatory cytokine network jointly maintain the balance between the proinflammatory and anti-inflammatory response systems, thereby safeguarding the normal physiological function of inflammation. The loss of physiological control of the inflammatory reaction is likely due to an imbalance of the finely tuned equilibrium between the levels of pro- and anti-inflammatory cytokines. When the balance is perturbed, this can lead to a chronic “proinflammatory” status, which promotes or exacerbates the above mentioned pathological conditions [31]. The final result of the counterbalance between pro- and anti-inflammatory cytokines (IL-4, IL-6, IL-13, and IL-10) is an upregulation of the proinflammatory response [35].

Studies of the functional effect(s) and mechanism of SIRT6 focus on three aspects [13, 36, 37]. Firstly, the influence of SIRT6 on the repair of DNA damage. Secondly, the influence of SIRT6 on aging and inflammation, where the key target is NF-*κ*B (p65). Thirdly, the influence of SIRT6 on the steady state that maintains the body's energy metabolism and where the key target is hypoxia-inducible factor 1 (Hif-1*α*).

To date, there is no evidence that ICA can affect NF-*κ*B (p65), a key regulator of inflammation, by affecting SIRT6, an important enzyme in aging regulation and control. In this study we provided experimental evidence for a direct, or indirect, interaction between ICA, SIRT6, and NF-*κ*B. We showed that ICA can upregulate SIRT6 protein expression and downregulate NF-*κ*B (p65) protein expression in animal tissues and cell models.

We provided evidence that, following treatment of aortic ECs with TNF-*α*, the mRNA expression level of SIRT6 was increased. Treatment with TNF-*α* and ICA however resulted in a significant increase in SIRT6 mRNA relative to TNF-*α* treatment alone. TNF-*α* and ICA treated cells subsequently treated with PDTC (NF-*κ*B inhibitor), Nicotinamide (SIRT6 inhibitor), or PDTC and Nicotinamide showed a trend of decreasing SIRT6 mRNA levels but these decreases were not significant when compared with TNF-*α* and ICA treatment (Figure 6(a)). Although these data support that ICA upregulates SIRT6, the effects on SIRT6 and NF-*κ*B, could be indirect.

We showed that treatment of young aortic ECs with the proinflammatory cytokine TNF-*α* resulted in increased levels of inflammatory cytokines that are downstream targets of NF-*κ*B (TNF-*α*, ICAM-1, IL-2, and IL-6), with TNF-*α* mRNA levels showing the greatest increase (Figure 6(b)). ICA intervention resulted in significantly decreased mRNA levels of all four inflammatory cytokines. Addition of PDTC to inhibit NF-*κ*B resulted in a further reduction in the expression of the four inflammatory cytokines but the reduction was not significant. These data suggest ICA can act as an inhibitor of NF-*κ*B. Treatment with TNF-*α*, ICA and the SIRT6 inhibitor Nicotinamide partially reversed the effects of ICA with respect to TNF-*α* and ICAM-1 mRNA levels. These data suggest that ICA can act as an agonist of SIRT6. Treatment with ICA and PDTC and Nicotinamide increased the expression of all four proinflammatory cytokines when compared with ICA and PDTC treatment. Treatment with ICA and PDTC and Nicotinamide completely reversed the effects of ICA on ICAM-1 mRNA levels and substantially reduced the effects of ICA on TNF-*α*, IL-2, and IL-6 mRNA levels. These data suggest that ICA can act through either the NF-*κ*B and SIRT6 pathways to regulate the expression of inflammatory cytokines. The synergistic effect suggests that ICA can affect the inflammation signal pathway of NF-*κ*B through SIRT6.

## 5. Conclusion

We conclude that ICA had a stimulatory effect on SIRT6 enzyme activity* in vitro*, even at very low concentrations. ICA can upregulate SIRT6 protein expression and downregulate NF-*κ*B (p65) protein expression in murine heart tissues and in an aortic EC model. ICA upregulated the expression of SIRT6 and had an inhibitory effect on NF-*κ*B inflammatory signaling pathways by decreasing the mRNA levels of NF-*κ*B downstream target genes TNF-*α*, ICAM-1, IL-2, and IL-6. Those effects were mediated directly or indirectly by SIRT6. We have provided tentative evidence that inflammaging may involve a link between the effects of ICA on SIRT6 (a regulator of aging) and NF-*κ*B (a regulator of inflammation).
